# Role descriptions induce gender mismatch effects in eye movements during reading

**DOI:** 10.3389/fpsyg.2015.01607

**Published:** 2015-11-03

**Authors:** Chiara Reali, Yulia Esaulova, Anton Öttl, Lisa von Stockhausen

**Affiliations:** ^1^Department of Psychology, University of Duisburg-EssenEssen, Germany; ^2^Department of Psychology, Norwegian University of Science and TechnologyTrondheim, Norway

**Keywords:** gender typicality, gender stereotypes, eye-tracking, sentence reading, anaphor resolution

## Abstract

The present eye-tracking study investigates the effect of gender typicality on the resolution of anaphoric personal pronouns in English. Participants read descriptions of a person performing a typically male, typically female or gender-neutral occupational activity. The description was followed by an anaphoric reference (*he* or *she*) which revealed the referent's gender. The first experiment presented roles which were highly typical for men (e.g., blacksmith) or for women (e.g., beautician), the second experiment presented role descriptions with a moderate degree of gender typicality (e.g., psychologist, lawyer). Results revealed a gender mismatch effect in early and late measures in the first experiment and in early stages in the second experiment. Moreover, eye-movement data for highly typical roles correlated with explicit typicality ratings. The results are discussed from a cross-linguistic perspective, comparing natural gender languages and grammatical gender languages. An interpretation of the cognitive representation of typicality beliefs is proposed.

## Introduction

In talking about human beings, gender information can be transmitted in different ways, e.g., via grammatical gender cues and gender-typical lexemes. Grammatical gender is marked, for example, in morphological elements which may express the gender of the referent such as the suffix *-in* in German (e.g., *Lehrer-in*, teacher_feminine_). The gender typicality of lexemes results from the likelihood of personal nouns to refer to men or women. Thus, the noun *nurse* has female typicality and *surgeon* male typicality, because of their likelihood to be associated with a female or a male referent respectively, as shown in typicality ratings (cf. Kennison and Trofe, 2003). The purpose of the present paper is to analyze the effect of gender typicality on the resolution of a pronominal anaphor when gender typicality is conveyed by a description of a role rather than a role noun antecedent. Namely, we investigate a socio-psychological concept, expectations about gender roles, with the help of a psycholinguistic tool, the paradigm of anaphor resolution during sentence reading. Our approach makes use of verbal descriptions and allows for comparing a natural gender language with a grammatical gender language, as will be outlined in detail below. The present study deals with English, a language which does not possess a grammatical gender system (“natural gender language,” see Hellinger and Bußmann, 2001). Since most professional roles lie in the range of moderate stereotypicality, we explore both the effect of roles with high and moderate degrees of gender typicality. Previous studies, however, mainly focused on the gender typicality effect of strongly stereotyped roles; thus, in a reading time study employing role nouns, Kennison and Trofe (2003) presented gender-typical roles as antecedents and personal pronouns as anaphors. The gender mismatch condition (e.g., *The executive…She…*) prompted longer reading times in the spillover region following the pronoun compared to the matching condition. The results indicated that the role nouns triggered gender-typical representations of the referent which either agreed or disagreed with the following pronominal anaphor.

Garnham et al. (2002) conducted a reading study employing both role nouns and short expressions referring to gender typical habits or biological characteristics (e.g., wearing a bikini; giving birth). The study shows that a mismatch between the two pieces of information produced longer reading times, even when the presentation order of the two pieces of information was reversed, suggesting that gender inferences were made elaboratively and not only when the inference was necessary for the coherent interpretation of the text.

In a reaction time study, Oakhill et al. (2005) asked participants to judge if pairs composed of gender stereotypical and gender definitional role nouns (e.g., *surgeon-sister*) could apply to the same person. Results showed that the activation of stereotypical information was automatic and difficult to suppress, even with instructions encouraging participants to explicitly reconsider the stereotypical representations of the roles.

Pyykkönen et al. (2010) explored the effect of gender stereotypes on spoken language processing in Finnish, a language which also does not possess a grammatical gender system, by means of the visual-world paradigm. Participants heard stories presenting a gender typical role noun, in association with pictures of male or female characters. Results showed an activation of gender stereotypes triggered by the spoken role nouns, even if this activation was not needed to establish greater discourse coherence.

Most psycholinguistic studies investigating gender typicality effects on anaphor resolution in English (e.g., for eye-tracking methodology Sturt, 2003; Duffy and Keir, 2004; Kreiner et al., 2008; for ERP methodology, Osterhout and Mobley, 1995; Osterhout et al., 1997) used reflexive pronouns (*himself/herself*) to reveal referential gender. The results of these studies document a consistent mismatch effect on the anaphor region or the subsequent region, caused by conflicts between the gender typicality of role noun antecedents and the following anaphors.

To summarize the main findings of studies on natural gender languages, one can state that incongruence between the gender typicality of the antecedent role nouns and the anaphor gender triggers a slowdown in resolution, for both personal and reflexive pronouns.

In grammatical gender languages, in contrast to natural gender languages, role nouns carry additional grammatical gender cues, which also affect the representation of referential gender. As a consequence, the effect of grammatical gender and gender typicality usually appear in interaction, and the specific contribution of the different factors can be difficult to disentangle.

Esaulova et al. (2014), for example, analyzed anaphor resolution after role nouns carrying both grammatical gender cues and gender typicality in an eye-tracking study on German, (e.g., *Oft hatte der Elektriker/die Elektrikerin gute Einfälle, regelmäßig plante er/sie neue Projekte*. “Often had the electrician_masculine∕feminine_ good ideas, regularly planned he/she new projects.”). In the condition of a mismatch between grammatical gender and gender typicality of the role noun results showed a mismatch effect not only on the anaphor region but also on the role noun region. The antecedent contained grammatical gender markings (either masculine or feminine ones), therefore the effect of the noun's gender typicality on anaphor resolution resulted from a combined processing of grammatical gender cues and typicality (see also Gygax et al., 2008; Irmen and Schumann, 2011).

A series of experiments conducted by Jäger et al. (2015), analyzed the online processing of reflexives in German and pronominal possessives in Swedish, by means of self-paced reading and eye-tracking methodology. The study focused on grammatical gender, conveyed through gender markings on role nouns (in German) or proper names (in Swedish). Materials presented an antecedent and a distractor, which could match or mismatch in gender (masculine/feminine). In contrast to previous studies, the results of these experiments showed no evidence for an online similarity-interference effect triggered by a gender overlap between the competitor role nouns. Only offline response accuracy to the comprehension questions in the self-paced reading experiment showed that the similarity-interference might have produced misretrievals of the distractors. These results suggest that the previously reported interference effects in reflexive processing may arise at the stage of retrieval rather than at the encoding stage.

The interplay of grammatical gender and gender typicality was further explored in a reading study on another grammatical gender language (Italian): Cacciari et al. (2011) investigated the resolution of personal pronouns in interaction with gender typicality. In the first part of each item, gender typicality was established through a context which described a typically male, female or neutral setting, for example “During the last Grand Prix of Formula One a terrible car accident provoked a crash close to the stands” (typically male context), or “Within the couple, scenes of jealousy were frequent but this time they came to blows and they got close to tragedy” (typically female context). In the second part of the item an epicene (a noun with a defined grammatical gender, but which can refer to both a male or female referent, e.g., *vittima*, male or female victim_feminine_) or a bigender role noun (a noun which can function both as a feminine and a masculine noun, e.g., *assistente*, assistant) was introduced as antecedent for an anaphoric pronoun. The anaphor could match or mismatch the typical context and/or the grammatical gender of the epicene. Results showed that for bigender role nouns, which did not present a defined grammatical gender, the influence of gender typicality was essential to trigger the mismatch effect; however, when the antecedent was an epicene the grammatical gender of the role noun, even though purely formal, affected the resolution of the anaphor and interfered with the typicality effect.

The reviewed literature shows that role nouns can represent a useful tool to convey and investigate gender typicality. However, role nouns can preclude a direct comparison of natural gender languages and grammatical gender languages, because in grammatical gender languages personal role nouns are usually marked for grammatical gender and therefore carry an additional cue to referential gender, whereas in natural gender languages most role nouns are not morphologically marked. This causes different processes in the resolution of anaphors with role noun antecedents, for in grammatical gender languages readers are presented both with grammatical information and information from gender typicality, while natural gender languages mostly present only cues from gender typicality. The complex interaction between grammatical cues and gender typicality represents a challenge in investigating effects of gender typicality, since the grammatical gender of role nouns may compete with gender typicality cues in the representation of referent gender. To overcome this issue, the present study employs a paradigm which replaces role nouns with corresponding role descriptions, in order to convey the gender typicality of a role without presenting the role noun itself. In a study by Reali et al. (2015), a description-based paradigm was developed to study the effect of gender typicality on anaphor resolution in a grammatical gender language, while excluding grammatical cues of the antecedents. This research raised a further research question, namely a cross-linguistic comparison of cognitive processes occurring in a “naturalized” grammatical gender language (i.e., a grammatical gender language without grammatical gender cues) and those in a natural gender language. Even in the absence of grammatical gender cues in the materials, speakers of a grammatical gender language may process gender typicality cues differently from speakers of a language without grammatical gender. Evidence from studies with bilinguals suggests that readers may activate different cognitive representations of referent gender according to the language of the task they are engaged in, shifting gender representations when switching from a natural gender language to a grammatical gender language and vice versa (see Sato et al., 2013). Starting from these considerations, the present study analyzes the processing of gender typicality in a natural gender language and compares the resolution process with previous studies conducted on a grammatical gender language (cf. Reali et al., 2015).

Another research question concerns the degree of gender typicality of the items. Earlier studies employing the anaphor resolution paradigm usually relied on highly typical roles and thus excluded the majority of social and professional roles, which do not occupy extreme positions on the gender typicality scale. Therefore, the second experiment of the present paper focuses on effects triggered by roles with lower degrees of gender typicality and examines if role descriptions with moderate degrees of gender typicality are able to elicit expectations in the referent gender representation, thus producing a disruption in the reading process when the mismatching pronoun is encountered.

The present research employs the methodology of eye-tracking, which provides high spatial and temporal resolution in mapping the process of anaphor resolution during reading.

## Experiment 1

The aim of Experiment 1 was to analyze the effect of gender typicality on pronominal anaphor resolution with a description-based paradigm. Specifically, the paradigm employed descriptions of gender-typical occupational roles instead of role nouns to convey gender typicality. The absence of role nouns allows us to compare the processing of gender typicality cues in natural gender and grammatical gender languages.

### Method

#### Participants

Thirty-one students (17 women and 14 men) from the University of Sussex, UK, participated in the study. Participants were English native speakers, with normal or corrected-to-normal vision (mean age = 21 years, *SD* = 3.9). They received monetary compensation or course credit for their participation. Ethical approval for the study was granted by the University of Sussex's Research Ethics Committee and all participants provided written informed consent before taking part in the study.

#### Design and hypothesis

The experiment was designed to test the interaction between the gender typicality of the occupational role (*typicality*: male, female, or neutral) and the gender of the anaphoric reference (*pronoun*: masculine or feminine). In accord with the German study (Reali et al., 2015) and earlier research using gender-typical role nouns, we expected a mismatch between gender-typical role description and anaphor gender to evoke longer fixation times and more frequent regressions compared to the matching and neutral conditions.

#### Materials

Materials were created to provide gender-typical information associated with different occupational activities without employing role nouns. The experimental sentences are based on the material of a study which had been conducted in German (Reali et al., 2015). In this previous study, a list of roles had been first selected from published collections of role nouns gender typicality ratings for different languages (Kennison and Trofe, 2003; Irmen, 2007; Gabriel et al., 2008). Then participants (30 women, 20 men, mean age = 23.1, *SD* = 4.1, students from the University of Heidelberg, Germany) estimated to which extent a specific professional role (e.g., primary school teacher) was held by men and/or women, using a 7-point scale with anchor points 1 = only men, 7 = only women, and 4 = same amount of women and men. Items (*N* = 77) were categorized as follows: male: ≤ 2.5, neutral: 3.5–4.5, female: ≥5.5. The same sample provided, through a written computer-based production task, a description of each role, on which the experimental items were based. These descriptions were then presented, in a paper-based questionnaire, to a new participant sample (*N* = 40, students from the University of Heidelberg), which had to guess the role nouns corresponding to the descriptions. This sub-test had the goal to check the correspondence between the role representation conveyed by the descriptions and the corresponding role nouns. Descriptions presenting less than 80% description-noun correspondence were discarded. This selection yielded 12 female, 12 male, and 12 neutral descriptions, to constitute the final material of 36 experimental items for the eye-tracking study. The last participant sample also rated the typicality of the final descriptions, which presented a strong correlation with the role noun rating (*r* = 0.995, *p* < 0.001). The differences between the three typicality conditions, calculated on the description typicality ratings (*M*_male_ = 1.87, *SD* = 0.42, *M*_female_ = 5.98, *SD* = 0.37, *M*_neutral_ = 4.17, *SD* = 0.37) were statistically significant, male–female: *t*_(22)_ = −30.23, *p* < 0.001; male–neutral: *t*_(22)_ = −20.24, *p* < 0.001; female–neutral: *t*_(22)_ = −18.99, *p* < 0.001. The pre-test procedure was fully conducted at the University of Heidelberg, Germany (see Reali et al., 2015). The resulting experimental material was translated and adapted to be employed for the present eye-tracking study.

Each experimental sentence consisted of a first part which described an occupation (“context”), and a second part containing a pronominal anaphor (“target sentence”). The personal pronoun (“he”/“she”) referred back to the person presented in the previous context, which had been introduced with initials, as in examples (1) (male typicality), and (2) (female typicality):

1. K. L. installs power lines and cables, checks electricity voltage.
  In this field he/she has a lot of experience.
2. L. K. teaches at a primary school, instructs children in reading.
  At work he/she wears thick glasses.

The gender neutrality of the target sentences had been ensured through a rating pre-test. In order to keep the anaphoric pronoun in a comparable position across items, all target sentences had a fixed linguistic structure, with the anaphor positioned between an initial adverbial expression and the verb.

In addition to the experimental sentences we presented 50 filler sentences containing descriptions of non-professional roles (e.g., moviegoer) and anaphoric expressions referring back to an inanimate object, to avoid drawing attention to the gender topic. Finally, we presented 24 content-related questions (e.g., “Is the lab coat green?”) in order to promote attentive reading, leading to a total number of 110 trials (including experimental items, fillers and questions).

#### Procedure

Eye movements were monitored with a video-based head mounted eye-tracker (Eyelink II, sampling rate of 250 Hz, average accuracy 0.5°). Materials were presented with the software Eyetrack^1^ on a 21-inch CRT computer screen, with an active screen size of 40 × 30 centimeters and a resolution of 1024 × 768 pixels. Participants were seated 70 cm away from the screen, at which distance 3 characters subtended approximately 1° of visual arc. A chinrest was used to minimize head movements. Reading was binocular but only the dominant eye was tracked. The dominant eye was determined through the Miles test^2^. The experiment began after a calibration procedure which was performed on a nine-point grid.

The presentation of sentences started with a small rectangle indicating the position of the first word of the sentence. The item appeared when the rectangle was fixated accurately. Whenever, the fixation on the rectangle was judged as inaccurate, re-calibration was carried out.

To familiarize participants with the task, the experiment started with four practice trials, one of which was followed by a comprehension question. Then the experimental sentences and filler items were presented. Sentences were displayed in a monospaced 22-point Lucida Console font, in black characters on a light gray background and consisted of three lines, presenting a maximum number of 49 characters each. The first two lines contained the role description; the third line presented the target sentence with the anaphoric reference. Experimental items were presented in randomized order across participants. After reading an item, participants pressed a button on a keypad to prompt the next item or a question. Two buttons of the keypad were used for answering the comprehension questions.

As a follow-up procedure, participants completed a questionnaire asking for gender typicality ratings, on a 7-point Likert scale, concerning the job descriptions that were presented in the eye-tracking session. The experiment lasted in total approximately 30–45 min.

### Results

#### Data analysis

In order to investigate the effect of the priming context on the target sentence, we analyzed fixation times and regression patterns on different regions of the target sentences. The target sentence was divided into four regions of analysis: adverb region, anaphor region, spillover region, and final region. The segmentation into regions of analysis is shown in Table 1.

**Table 1:** **Experiment 1 factorial structure and regions of analyses (delimited by a dash)**.

Context	Male role description	C. R. repairs and produces furniture, works with wood.
	Female role description	K. P. sells flowers, makes up bouquets in a shop.
	Neutral role description	F. H. plays an instrument professionally in an orchestra.
Target	Anaphoric reference	Usually - he/she has - a sufficient - income. Reg.1 Reg.2 Reg.3 Reg.4

In order to reflect the processing of the text from early to late stages, data were analyzed for the following eye-tracking measures: first fixation time, first pass time, regression path time, total time, and probabilities of regressions into and out of a region. First fixation time is the duration of the first fixation in a given region. First pass time is the time from first entering a region of interest from the left until leaving it either to the right (i.e., moving forward in the sentence) or to the left. Regression path is the time from first entering a region until leaving it to the right, including the time for regressions from this region. Total time is the total amount of time spent in a certain region including re-reading, but not including regressions from this region. Regressions into and out of a region, respectively, consist of the proportion of backward movements into a specific region, or leaving the region to the left after a first pass fixation of the region (cf. Sturt, 2003; Boland, 2004). In general, longer fixation times and a higher probability of regressions are indicative of greater difficulty in processing the respective region.

Initial stages of data analysis were carried out using the software EyeDoctor and EyeDry provided by the Department of Psychology at the University of Massachusetts Amherst. Short fixations (below 70 ms) were merged with neighboring fixations within three characters. Following Reali et al. (2015), we removed fixations below 70 ms and above 600 ms, as they can be assumed to be not representative of regular information acquisition during reading (4.1% of the data). The remaining data have been logarithmically transformed to meet the normality assumption for the following analyses. No significant difference emerged in the distribution of missing data across typicality conditions for all regions and fixation duration measures [*M*_male_ = 74.00; *M*_female_ = 74.19; *M*_neutral_ = 69.06, *F*_(2, 45)_ = 0.86, *ns*]. Analyses were based on linear mixed-effect modeling, implemented by the *lmer* function from the lme4 package (Bates et al., 2014) in R (R Core Team, 2012, version 2.15.2). We included in our models participants and items as random effects (see Baayen et al., 2008). As fixed effects for our models we selected the experimental factors that were assumed to influence the target sentence processing: *gender typicality* of the priming sentence (male, female, or neutral) and *pronoun* of the target sentence (masculine, feminine). In addition, we included *region length* (number of characters for each region of analysis) in all fixation duration measures (i.e., excluding regression measures), and *participant gender*, as fixed effects, since these factors could affect the reading processes, Model <- lmer [fixation_time ~ typicality ^*^ pronoun ^*^ participant_gender ^*^ region_length + (1 |participants) + (1 |items)].

To systematically detect the best fitting model for each measure and region, we employed the *step* function available in lmerTest package (Kuznetsova et al., 2013), which was developed with the purpose of automatizing and standardizing the model building process. Starting from a fully specified model, *step* performs a backward elimination of both random and fixed effects that are not warranted by the data by conducting iterative model comparisons. The function is based on likelihood ratio tests and step-wise removal of non-significant fixed effect terms. Significant effects of pronoun, typicality and their interaction were further explored through contrast analyses. Pairwise comparisons tested each typicality condition followed by masculine and feminine pronouns (male–he vs. male–she; female–he vs. female–she; neutral–he vs. neutral–she).

#### Eye-tracking results

The final models for each measure and region (including all significant random effects, fixed effects, and interactions) are reported in Supplementary Material (Table S1). Means and standard deviations of fixation duration time and percentages of regressions are reported in Table 2,^3^. Details on statistical results are reported in Table 3. We report below eye-tracking measures presenting statistically significant fixed effects of *typicality, pronoun*, and typicality*pronoun (*p* < 0.05), and corresponding significant or marginally significant (*p* < 0.1) results of contrast analyses, separated for measure.

**Table 2:** **Means (standard deviations) of fixation duration time (ms) and percentages of regressions for Experiment 1**.

**Region**	**Typ**.	**Pron**.	**Reg. 1**		**Reg. 2**		**Reg. 3**		**Reg. 4**	
FF	Male	He	202	(59)	191	(66)	205	(79)	216	(94)
		She	214	(82)	192	(67)	204	(66)	230	(106)
	Fem.	He	209	(68)	198	(75)	215	(87)	232	(111)
		She	205	(76)	184	(66)	207	(73)	237	(108)
	Neutr.	He	197	(61)	198	(62)	211	(81)	233	(111)
		She	196	(63)	188	(64)	217	(80)	224	(105)
FP	Male	He	245	(85)	254	(140)	313	(182)	340	(282)
		She	253	(98)	272	(176)	328	(203)	334	(242)
	Fem.	He	246	(108)	270	(165)	348	(203)	340	(253)
		She	233	(84)	266	(159)	307	(204)	339	(236)
	Neutr.	He	235	(82)	248	(114)	316	(244)	295	(192)
		She	226	(68)	255	(137)	327	(216)	322	(236)
RP	Male	He	265	(164)	372	(312)	585	(681)	950	(776)
		She	290	(187)	388	(335)	538	(496)	1047	(869)
	Fem.	He	246	(108)	369	(270)	563	(422)	1096	(877)
		She	246	(126)	347	(191)	496	(380)	1093	(969)
	Neutr.	He	243	(110)	325	(232)	680	(618)	901	(828)
		She	243	(121)	306	(202)	629	(719)	973	(815)
TT	Male	He	275	(139)	384	(239)	456	(294)	427	(322)
		She	295	(170)	406	(255)	439	(267)	412	(270)
	Fem.	He	275	(146)	416	(224)	497	(299)	466	(330)
		she	261	(130)	389	(227)	459	(297)	428	(281)
	Neutr.	He	279	(139)	389	(207)	512	(390)	371	(245)
		She	264	(118)	395	(260)	501	(319)	393	(267)
RI	Male	He	28	(45)	30	(46)	22	(42)	–	–
		She	22	(41)	32	(47)	22	(42)	–	–
	Fem.	He	26	(44)	35	(48)	26	(44)	–	–
		She	30	(46)	30	(46)	22	(42)	–	–
	Neutr.	He	22	(42)	44	(50)	20	(40)	–	–
		She	21	(41)	42	(49)	20	(40)	–	–
RO	Male	He	2	(15)	21	(41)	30	(46)	47	(50)
		She	4	(19)	17	(38)	25	(43)	53	(50)
	Fem.	He	0	(0)	19	(40)	32	(47)	59	(49)
		She	2	(13)	19	(40)	30	(46)	52	(50)
	Neutr.	He	1	(10)	13	(34)	42	(49)	45	(50)
		She	2	(13)	9	(29)	35	(48)	56	(50)

FF, first fixation time; FP, first pass time; RP, regression path; TT, total time; RI, regressions into the region; RO, regressions out of the region.

**Table 3:** **Statistical results for Experiment 1**.

	**First fixation time**		**First pass time**		**Total time**	
	**(DF) *F*-value**	**Pr (>F)**	**(DF) *F*-value**	**Pr (>F)**	**(DF) *F*-value**	**Pr (>F)**
**FIRST REGION**						
Pronoun	(1690) 0.332	0.564	(1668) 0.072	0.789	(1875) 0.524	0.469
Typicality	(2691) 5.655	0.003^*^	(242) 1.557	0.223	(232) 1.120	0.339
Pron. ^*^ Typ.	(2697) 0.406	0.666	(2674) 1.662	0.190	(2873) 1.031	0.357
**PRONOUN REGION**						
Pronoun	(1892) 2.842	0.092	(1883) 0.522	0.470	(11,008) 1.134	0.287
Typicality	(2888) 0.349	0.706	(2883) 0.131	0.877	(235) 0.165	0.848
Pron. ^*^ Typ.	(2886) 1.571	0.208	(2883) 0.435	0.647	(21,016) 2.003	0.136
**SPILLOVER REGION**						
Pronoun	(1958) 0.022	0.883	(1948) 0.055	0.816	(11,011) 1.265	0.261
Typicality	(232) 0.521	0.599	(232) 0.551	0.582	(231) 0.143	0.867
Pron. ^*^ Typ.	(2955) 0.578	0.561	(2948) 4.442	0.012^*^	(21,003) 3.015	0.049^*^
**FINAL REGION**						
Pronoun	(1795) 0.324	0.569	(1761) 0.521	0.471	(1773) 0.008	0.928
Typicality	(2799) 0.596	0.551	(231) 0.130	0.879	(232) 0.255	0.776
Pron. ^*^ Typ.	(2793) 0.469	0.626	(2755) 0.197	0.821	(2765) 0.167	0.846
	**Regression path**		**Regressions in**		**Regressions out**	
**FIRST REGION**						
Pronoun	(1678) 0.046	0.830	(11,043) 0.282	0.595	(11,082) 2.714	0.100
Typicality	(231) 2.418	0.105	(233) 0.939	0.401	(21,083) 2.876	0.057
Pron. ^*^ Typ.	(2677) 0.628	0.534	(21,043) 1.176	0.308	(21,077) 0.222	0.801
**PRONOUN REGION**						
Pronoun	(1886) 7.491	0.006^*^	(11,048) 1.092	0.296	(11,042) 1.646	0.199
Typicality	(233) 0.781	0.466	(233) 2.705	0.082	(233) 2.184	0.128
Pron. ^*^ Typ.	(2855) 0.360	0.698	(21,045) 0.752	0.472	(21,042) 0.148	0.862
**SPILLOVER REGION**						
Pronoun	(1941) 4.594	0.032^*^	(11,050) 0.206	0.650	(11,049) 3.713	0.054
Typicality	(232) 1.055.	0.358	(233) 0.266	0.768	(233) 1.180	0.320
Pron. ^*^ Typ.	(2938) 0.805	0.447	(21,042) 0.321	0.726	(21,046) 0.216	0.806
**FINAL REGION**						
Pronoun	(1762) 0.486	0.486	–	–	(11,047) 1.608	0.205
Typicality	(2757) 1.514	0.221	–	–	(233) 0.392	0.679
Pron. ^*^ Typ.	(2755) 0.324	0.723	–	–	(21,047) 3.363	0.035^*^

Significance codes:

* p < 0.05.

##### First pass time

The first reliable interaction effect between *typicality* and *pronoun* was detected in first pass time on the region immediately following the pronoun (spillover)^4^. Contrast analyses revealed that the effect was statistically significant only when the priming sentence was female, with congruent trials being read faster, *M*_femaleHE_ = 302, *M*_femaleSHE_ = 263, *t*_(948)_ = 2.55, *p* = 0.01; *M*_maleHE_ = 257, *M*_maleSHE_ = 269, *ns*; *M*_neutralHE_ = 269, *M*_neutralSHE_ = 288, *ns*.

##### Regression path time

A main effect of *pronoun* appeared on the pronoun region and on the spillover. Contrast analyses showed that the feminine pronoun condition was read faster, *M*_HE_ = 295, *M*_SHE_ = 269, *t*_(514)_ = 2.35, *p* = 0.002 (pronoun region); *M*_HE_ = 457, *M*_SHE_ = 407, *t*_(941)_ = 2.14, *p* = 0.03 (spillover region).

##### Regressions out of a region

The interaction between *typicality* and *pronoun* emerged in the proportion of regressions out of the last region of the target sentence. Contrast analyses showed a significant effect for the neutral condition, presenting less regressions in association with a masculine as compared to a feminine pronoun, *M*_neutralHE_ = 8.1, *M*_neutralSHE_ = 13.2, *t*_(947)_ = −2.26, *p* = 0.02; *M*_maleHE_ = 8.9, *M*_maleSHE_ = 11.7, *ns*; *M*_femaleHE_ = 14.8, *M*_femaleSHE_ = 11.2, *ns*.

##### Total fixation time

The interaction between *typicality* and *pronoun* emerged on the spillover region. Pairwise comparisons revealed a significant effect for the female condition, but not for the male and neutral conditions, with shorter fixation time on congruent trials as compared to incongruent ones, *M*_femaleSHE_ = 380, *M*_femaleHE_ = 427, *t*_(998)_ = 2.14, *p* = 0.03; *M*_maleHE_ = 363, *M*_maleSHE_ = 355, *ns*.; *M*_neutralHE_ = 437, *M*_neutralSHE_ = 437, *ns.* Furthermore, a main effect of *participant gender* emerged on the pronoun region. Contrasts revealed a tendency for female participants to read faster, *M*_men_ = 355, *M*_women_ = 316, *t*_(30)_ = 1.86, *p* = 0.073.

#### Gender typicality ratings and eye movements

Typicality, ratings for Experiment 1 are reported in Supplementary Material (Table S2). Typicality ratings were based on the data collected in a previous study (see Materials section), from a sample which did not participate in the eye-tracking experiment. In order to investigate if eye movements reflected the extent of gender expectations, we conducted a by-item linear regression analysis with typicality ratings as predictors of eye movements. We selected the regions of analysis where the gender mismatch effect emerged. Since pairwise comparisons revealed an asymmetry between the male and female condition, we conducted separate analyses for the two anaphoric pronouns. Results revealed that typicality ratings predicted first pass fixation times after a masculine anaphor (β = 0.35, *p* < 0.05). As the scale for typicality ratings presented the poles 1 = male, and 7 = female, the β coefficient showed a direct correlation in the condition of the masculine pronoun, with lower ratings predicting shorter fixations after the pronoun *he*. This result indicates that fixation time on a region where the mismatch effect emerged corresponded to the degree of gender typicality expressed in the explicit typicality ratings of the respective items.

#### Follow-up typicality ratings

Follow-up typicality ratings were collected from participants immediately after completing the eye-tracking experiment. The follow-up ratings showed a high correlation with the pre-test ratings (*r* = 0.966, *p* < 0.001). However, male and female typicality turned out to be more skewed toward neutrality, so that typically male and particularly typically female occupations received less extreme ratings as compared to the pretest ratings, *M*_male_, pretest = 1.87, *M*_male_, follow-up = 2.32, *t*_(22)_ = 2.88, *p* = 0.009; *M*_female_, pretest = 5.98, *M*_female_, follow-up = 5.20, *t*_(22)_ = 4.20, *p* < 0.001; *M*_neutral_, pretest = 4.04, *M*_neutral_, follow-up = 4.16, *t*_(22)_ = 0.85, *ns*.

### Discussion

The study analyzed the effect of gender typicality cues on the resolution of a pronominal anaphor. As antecedents, the commonly used role nouns were replaced with role descriptions which contained only gender typicality cues to referent gender. The experiment was conducted in English, a language which does not possess a grammatical gender system.

A main effect of pronoun emerged in regression path on the pronoun and spillover region, with the feminine pronoun receiving shorter fixation time than the masculine pronoun. This effect may suggest a general greater difficulty to integrate a male as compared to a female referent. However, it should be noted that this effect is limited to this time measure, therefore representing an isolated finding rather than a systematic pattern.

The interaction between gender typicality of the description and pronoun gender is in the focus of the study and emerged in measures representing different stages of processing. Results showed that a mismatch effect between the two factors occurred reliably in a measure of early processing on the region following the anaphoric pronoun. Moreover, this interaction was detected consistently in a measure of intermediate stage of processing (i.e., when participants regressed from the last region at the end of the target sentence to re-check the previously read sentence) and in one measure of late processing, namely the total amount of time spent on the pronoun spillover region. Furthermore, correlational analyses with gender typicality ratings showed that the typicality degree of the different items predicted the mismatch effect revealed by early fixation times, confirming the validity of the description paradigm as a tool to investigate gender typicality.

The location of the early mismatch effect is consistent with data from reading studies in English which employed role nouns as antecedents and personal pronouns as anaphors (Kennison and Trofe, 2003). The effect appears to be delayed in location and time in regard to studies employing reflexive pronouns to trigger the mismatch (e.g., Sturt, 2003). However, the effect cannot be compared directly because of relevant differences in sentence structure and paradigms used in the studies.

The present data can now be compared to a parallel study on German, where grammatical gender cues were avoided in the materials (Reali et al., 2015). Interestingly, in the German study the mismatch effect occurred earlier (in first fixations), on the pronoun region. Furthermore, in the German experiment the mismatch effect surfaced in two further measures (regressions in and total time) on the pronoun region itself. A possible explanation of the difference to the present findings concerns the presence or absence of grammatical gender in the two languages. The description-based paradigm served to keep the texts free of morphological gender cues in both languages. However, the processing of gender typicality cues may activate grammatical gender in the language with a grammatical gender system and thus cognitively facilitate the assignment of referent gender in the direction suggested by gender typicality. This would explain why the reference resolution process appears to be faster in the grammatical gender language. Previous eye-tracking studies using plural role nouns as antecedents also may support the interpretation that grammatical gender cues make gender typicality cues more salient and speed up the eventual gender mismatch effect. For example, in an eye-tracking experiment with German material, Irmen (2007) employed a noun phrase as anaphor (“these men/these women”). When antecedents were masculine generics, the typicality mismatch effect appeared on the first word of the anaphoric phrase itself in first pass reading (“these”). In contrast, when the antecedents had the form of gender-unmarked role nouns (e.g., *Alleinerziehende*, single parents) the typicality mismatch effect fully emerged only in later measures on the spillover region.

A further point of discussion is the asymmetry for the male and female condition, revealed in the pairwise comparisons of the mismatch effect. Specifically, gender mismatch was reliable only for the female condition, which produced an impairment in the sentence processing when followed by a masculine pronoun. This asymmetry was reliable in early and later stages of processing, on the target sentence spillover. The asymmetry effect may be interpreted as indicative of readers' difficulty to integrate a male referent with the representation of a typically female occupation; in contrast, reconciling a female referent with a typically male professional role apparently required less cognitive effort. Moreover, regressions launched from the last region show that the neutral condition may be integrated more easily with a masculine rather than a feminine anaphoric pronoun. This finding may represent a wrap-up effect emerging at the end of the sentence, after all the available information presented in the text had been collected. In this case, it may reflect a generally easier integration for the masculine as compared to the feminine referent when no specific gender cue is available, as in the case of neutral context.

Finally, follow-up typicality ratings, collected immediately after the eye-tracking session, showed less extreme ratings as compared to the pre-test ratings, for the male and particularly for the female condition. This finding is surprising since it was the female typicality that triggered the significant mismatch effect. In other words, participants found it particularly difficult to associate the representation of a male referent to a female occupation in the online measure, while the explicit ratings show that the female roles were judged as partially suitable also for men. We believe that participants may have been primed with counter-stereotypical representations of the roles through the recent exposure to the eye-tracking stimuli. While the present experiment was not designed to determine such a priming effect, it is plausible to suspect such an effect after a task where participants had to perform the cognitive task to integrate a stereotypical gender context with the gender incongruent referent. As shown by the eye movement data, this task may have been particularly surprising and consequently more salient for the female condition, thus priming later, on the offline ratings, a more equal representation of the gender distribution in the typical occupational roles.

## Experiment 2

Experiment 1 investigated the effect of typicality with the help of highly gender-typical items. However, the selection of such items excluded occupational roles in the range between gender-typical and neutral (see the Materials section for details). Therefore, the second experiment examines the following research question: Do occupational roles which are judged as slightly typical—but not as gender-neutral—affect the process of anaphor resolution? In other words, do readers develop a probabilistic cognitive expectation of referent gender when reading a description of roles with low gender typicality, such as *psychologist* or *lawyer*, which were rated as only *slightly* female and *slightly* male in the off-line measures?

### Method

#### Participants

Twenty-nine students (17 women and 12 men) from the University of Sussex, UK, participated in the study. Participants were native English speakers, with normal or corrected-to-normal vision (mean age = 21 years, *SD* = 2.4). None of them had participated in Experiment 1. They received monetary compensation or course credit for their participation. All participants provided written informed consent before taking part in the study.

#### Design and hypothesis

The experiment was designed to test the interaction between the gender typicality of the occupational role (*typicality*: slightly male, slightly female, or neutral) and the gender of the anaphoric reference (*pronoun*: masculine or feminine). If stimuli with moderate degrees of gender typicality can elicit expectations on the referent gender, then a disruption in the reading process would emerge when the mismatching pronoun is presented. This disruption would result in longer fixation times and higher probabilities of regressions. No effect is expected with neutral priming stimuli.

#### Materials

Item structure was identical to the one used in Experiment 1. In Experiment 2, the priming context was constituted of slightly male, slightly female, or neutral occupational roles. The selection of the roles was based on the role noun pretest (see Materials section, Experiment 1). We selected items with role noun typicality ratings between 2.5 and 3.5 (slightly male), 4.5 and 5.5 (slightly female) and 3.5 and 4.5 (neutral) on a 7-point Likert scale for gender typicality, where 1 represented the pole of male and 7 the pole of female typicality (*M*_s.male_ = 2.99, *SD* = 0.16, *M*_s.female_ = 4.98, *SD* = 0.31, *M*_neutral_ = 4.04, *SD* = 0.14). (3) and (4) are examples of a slightly male (3) and a slightly female (4) experimental item:

(3) C. H. earned a degree in law after many years of study.
  Nowadays he/she does mostly paperwork.
(4) H. C. receives calls from many customers at the call-center.
  Regularly he/she takes short breaks.

Participants were presented with 12 slightly male, 12 slightly female, and 12 neutral role descriptions. In addition, we randomly presented 50 filler sentences (the same items as in Experiment 1), and 24 content-related questions to promote attentive reading.

#### Procedure and analysis

The experimental procedure with eye-tracking recordings and the analyses were identical to those in Experiment 1. No significant difference emerged in the distribution of missing data across typicality conditions for all regions and fixation duration measures [*M*_s.male_ = 42.00; *M*_s.female_ = 35.00; *M*_neutral_ = 46.88, *F*_(2, 45)_ = 1.01, *ns*]. The mixed-effect models included participants and items as random effects. As fixed effects we included *typicality* (slightly male, slightly female, neutral), *pronoun* (masculine, feminine), *region length* (in fixation duration measures) and *participant gender*, Model <- lmer(fixation_time ~ typicality ^*^ pronoun ^*^ participant_gender ^*^ region_length + (1 |participants) + (1 |items).

### Results

#### Eye-tracking results

The final models for each measure and region (including all significant random effects, fixed effects, and interactions) are reported in Supplementary Material (Table S1). Means and standard deviations of fixation duration time and percentages of regressions are reported in Table 4. Details on statistical results are reported in Table 5. We report below eye-tracking measures presenting statistically significant fixed effects of *typicality, pronoun*, and typicality*pronoun (*p* < 0.05), and corresponding significant or marginally significant (*p* < 0.1) results of contrast analyses, separated for measure. Contrast analyses tested each typicality condition followed by the masculine and feminine pronoun (slightly male–he vs. slightly male–she; slightly female–he vs. slightly female–she; neutral–he vs. neutral–she).

**Table 4:** **Means (standard deviations) of fixation duration time (ms) and percentages of regressions for Experiment 2**.

**Region**	**Typ**.	**Pron**.	**Reg. 1**		**Reg. 2**		**Reg. 3**		**Reg. 4**	
FF	Male	He	208	(74)	195	(58)	207	(70)	240	(104)
		She	211	(74)	195	(57)	202	(67)	232	(94)
	Fem.	He	209	(78)	198	(58)	222	(79)	230	(95)
		She	216	(80)	202	(67)	220	(77)	222	(84)
	Neutr.	He	207	(71)	211	(78)	212	(84)	219	(207)
		She	196	(58)	196	(69)	218	(79)	217	(196)
FP	Male	He	237	(87)	249	(117)	300	(142)	347	(218)
		She	238	(91)	269	(128)	289	(154)	352	(234)
	Fem.	He	254	(107)	292	(141)	331	(148)	339	(254)
		She	251	(104)	315	(168)	325	(138)	369	(251)
	Neutr.	He	250	(107)	278	(140)	336	(228)	310	(250)
		She	240	(87)	266	(140)	327	(166)	347	(240)
RP	Male	He	263	(246)	326	(230)	492	(365)	988	(719)
		She	256	(153)	354	(247)	484	(430)	976	(790)
	Fem.	He	267	(129)	357	(242)	536	(497)	912	(672)
		She	261	(140)	368	(245)	538	(439)	896	(646)
	Neutr.	He	280	(218)	318	(227)	687	(640)	796	(632)
		She	261	(147	331	(235)	638	(560)	916	(826)
TT	Male	He	294	(152	413	(232)	448	(238)	423	(245)
		She	323	(213	427	(292)	455	(266)	438	(276)
	Fem.	He	316	(190	454	(276)	450	(233)	415	(250)
		She	282	(142	450	(264)	447	(204)	436	(309)
	Neutr.	He	303	(145	425	(239)	495	(306)	359	(277)
		She	305	(170	419	(245)	485	(276)	397	(285)
RI	Male	He	19	(39)	37	(49)	24	(43)	–	–
		She	28	(45)	35	(48)	25	(44)	–	–
	Fem.	He	20	(40)	28	(45)	20	(40)	–	–
		She	10	(31)	29	(46)	20	(40)	–	–
	Neutr.	He	17	(37)	32	(47)	16	(37)	–	–
		She	18	(39)	36	(48)	17	(38)	–	–
RO	Male	He	1	(11)	13	(34)	30	(46)	57	(50)
		She	2	(13)	14	(35)	25	(44)	57	(50)
	Fem.	He	2	(13)	10	(31)	25	(44)	52	(49)
		She	2	(13)	7	(25)	27	(45)	53	(50)
	Neutr.	He	3	(17)	6	(23)	36	(48)	43	(50)
		She	3	(17)	10	(31)	36	(48)	43	(50)

FF, first fixation time; FP, first pass time; RP, regression path; TT, total time; RI, regressions into the region; RO, regressions out of the region.

**Table 5:** **Statistical results for Experiment 2**.

	**First fixation time**		**First pass time**		**Total time**	
	**(DF) *F*-value**	**Pr (>F)**	**(DF) *F*-value**	**Pr (>F)**	**(DF) *F*-value**	**Pr (>F)**
**FIRST REGION**						
Pronoun	(1861) 0.026	0.871	(1831) 0.225	0.635	(1895) 0.103	0.748
Typicality	(2857) 1.430	0.240	(239) 1.234	0.302	(238) 1.589	0.217
Pron. ^*^ Typ.	(2855) 1.315	0.269	(2828) 0.065	0.937	(2899) 0.054	0.948
**PRONOUN REGION**						
Pronoun	(1903) 2.399	0.122	(1878) 0.171	0.679	(1844) 2.970	0.085
Typicality	(2905) 6.839	0.001^**^	(2330) 0.486	0.620	(232) 1.550	0.228
Pron. ^*^ Typ.	(2898) 0.545	0.580	(227) 3.872	0.021^*^	(2923) 0.371	0.690
**SPILLOVER REGION**						
Pronoun	(1918) 0.009	0.923	(1761) 0.749	0.387	(1940) 0.001	0.981
Typicality	(232) 2.127	0.136	(232) 0.239	0.788	(230) 3.050	0.062
Pron. ^*^ Typ.	(2913) 0.968	0.380	(2760) 0.367	0.693	(2933) 0.106	0.899
**FINAL REGION**						
Pronoun	(1812) 0.655	0.418	(1761) 0.749	0.387	(1781) 1.500	0.221
Typicality	(2814) 1.725	0.179	(232) 0.239	0.789	(233) 0.928	0.405
Pron. ^*^ Typ.	(2808) 0.040	0.961	(2760) 0.367	0.692	(2780) 1.080	0.339
	**Regression path**		**Regressions in**		**Regressions out**	
**FIRST REGION**						
Pronoun	(1834) 0.171	0.680	(1978) 0.004	0.952	(10) 0.048	0.826
Typicality	(229) 0.165	0.848	(233) 1.628	0.212	(20) 1.014	0.363
Pron. ^*^ Typ.	(230) 0.038	0.963	(2978) 5.466	0.004^*^	(20) 0.048	0.952
**PRONOUN REGION**						
Pronoun	(1812) 0.024	0.877	(1980) 0.097	0.756	(1980) 0.211	0.646
Typicality	(233) 0.440	0.648	(233) 1.221	0.308	(233) 2.014	0.150
Pron. ^*^ Typ.	(2515) 0.324	0.723	(2975) 0.437	0.646	(2978) 1.757	0.173
**SPILLOVER REGION**						
Pronoun	(1903) 0.348	0.556	(1980) 0.049	0.824	(1978) 0.190	0.663
Typicality	(232) 1.772	0.186	(233) 1.670	0.204	(233) 1.682	0.202
Pron. ^*^ Typ.	(2900) 0.744	0.475	(2975) 0.053	0.948	(2976) 0.681	0.506
**FINAL REGION**						
Pronoun	(1767) 0.002	0.968	–	–	(1978) 0.037	0.847
Typicality	(2769) 2.562	0.078	–	–	(233) 3.461	0.043^*^
Pron. ^*^ Typ.	(2757) 0.379	0.684	–	–	(2975) 0.048	0.953

Significance codes:

“*” p < 0.05;

“**” p < 0.001.

##### First fixation time

A main effect of typicality emerged on the second region of the target sentence. Pairwise comparisons between all the factor levels showed no reliable difference, M_s.male_ = 191, M_s.female_ = 186, M_neutral_ = 186, *ns*.

##### First pass time

The interaction between typicality and pronoun emerged on the pronoun region. Pairwise comparisons, however, showed no significant effect, *M*_s.maleHE_ = 234, *M*_s.maleSHE_ = 245, *ns*; *M*_s.femaleHE_ = 240, *M*_s.femaleSHE_ = 257, *ns*; *M*_neutralHE_ = 251, *M*_neutralSHE_ = 257, *ns.*

##### Regressions into a region

The interaction between *typicality* and *pronoun* emerged in regressions in the first region of the target sentence. Contrast analyses showed a significant effect for the female priming condition, where the congruent trials presented fewer regressions as compared to the incongruent ones, *M*_s.femaleSHE_ = 1.6, *M*_s.femaleHE_ = 2.5, *t*_(978)_ = 2.48, *p* = 0.01. The effect was also significant for the male condition, with congruent trials presenting fewer regressions as compared to the incongruent ones, *M*_s.maleHE_ = 2.4, *M*_s.maleSHE_ = 3.5, *t*_(978)_ = −2.14, *p* = 0.03. No effect was found for the neutral priming condition, *M*_neutralHE_ = 2.1, *M*_neutralSHE_ = 2.3, *ns.*

##### Regressions out

Regressions out of the last region showed a main effect of typicality. Pairwise comparisons revealed a smaller proportion of regressions for the neutral condition as compared to the slightly male condition, *M*_s.male_ = 14.1, *M*_neutral_ = 7.2, *t*_(33)_ = −2.58, *p* = 0.01, as well as a tendency for the neutral condition to present fewer regressions as compared to the slightly female condition, *M*_s.female_ = 11.2 *M*_neutral_ = 7.2, *t*_(33)_ = −1.75, *p* = 0.09. Probability of regressions did not differ for female and male conditions, *M*_s.female_ = 11.2, *M*_s.male_ = 14.1, *ns*.

##### Total fixation time

A main effect of participant gender emerged on the pronoun region. Contrasts revealed no significant difference, *M*_men_ = 363, *M*_women_ = 355, *ns*.

#### Gender typicality ratings

Typicality ratings for Experiment 2 are reported in Supplementary Material (Table S3). Follow-up typicality ratings correlated with the pretest ratings of the role nouns (*r* = 0.827, *p* < 0.001). As a whole, follow-up typicality ratings did not differ from pre-test ratings, *M*_pretest_ = 4.0, *M*_follow−up_ = 4.1, *t*_(70)_ = 0.325, *ns*. When analyzed separately, male and female typicality turned out to be more skewed toward neutrality in the ratings collected after the eye-tracking experiment, *M*_s.male_, pretest = 2.99, *M*_s.male_, follow-up = 3.34, *t*_(22)_ = −2.86, *p* = 0.009; *M*_s.female_, pretest = 4.98, *M*_s.female_, follow-up = 4.68, *t*_(22)_ = 2.20, *p* = 0.039; *M*_neutral_, pretest = 4.04, *M*_neutral_, follow-up = 4.16, *t*_(22)_ = 1.07, *ns*.

The mismatch effect found in eye movements did not correlate with explicit typicality ratings (β 's ≤ 0.07).

#### Discussion

Experiment 2 documents an effect of slightly gender-typical roles on the resolution of mismatching anaphoric personal pronouns, manifest in an early to intermediate stage of sentence processing. As in Experiment 1, gender typicality cues were conveyed through sentences describing a professional activity. In this experiment the occupations had been rated as only slightly typical for men or women, or as neutral. Still, slightly typical contexts were able to trigger the mismatch effect, as opposed to neutral priming trials. When description typicality and pronoun gender mismatched, readers regressed to the beginning of the target sentence, in order to re-check information and eventually resolve the gender conflict. The description-paradigm proved to be sensitive, showing that low degrees of typicality may evoke an impairment in the resolution process, and may thus be considered an adequate tool for investigating gender typicality, even when typical gender cues are too subtle to be categorized as “stereotypical.”

Differently from Experiment 1, in Experiment 2 the mismatch effect emerged in relation to both gender priming contexts. This may be explained by the fact that the second experiment presented slightly typical contexts, which may not produce a specific difficulty for the integration of the two gender conditions, as in the case of the integration of male referents in highly stereotypical roles. In other words, in the second study both gender priming conditions produced a reading impairment, as opposed to the neutral priming condition, in which integration with the pronoun did not prove problematic.

## General discussion

The study presented a paradigm to investigate the effect of gender typicality on pronominal anaphor resolution without relying on role nouns as antecedents. Gender typicality was prompted through descriptions of occupational roles. Results showed that gender typicality was conveyed effectively, that it affected the process of anaphor resolution in both a condition of high (Experiment 1) and low (Experiment 2) degree of the priming gender context. Incongruence between gender typicality of the description and pronoun gender produced a mismatch cost, which was mainly located on the pronoun region and its immediate spillover for fixation duration measures, and at the beginning and ending of the target sentence for the regression measures. While in Experiment 1 the explicit ratings could predict eye movements, no correlation was found in Experiment 2.

Taken together, these results offer insight into the representational format of gender typicality beliefs. First, the results suggest that the cognitive process of correcting for and integrating the initial mismatching gender representation exhibited a different time course in the two experiments: a more complex repair strategy involving early and late stages of processing was applied in the case of highly typical items, whereas less typical items only affected an early to intermediate stage of sentence processing.

Second, the results suggest that the effect of gender typicality can have two different cognitive sources: gender typicality and gender stereotypes. Gender typicality refers to the cognitive representation of the proportion of men and women in certain occupational roles and can be measured through explicit ratings. Gender stereotypes are cognitive representations which associate an occupational role with a specific gender and may be implicit, i.e., may not be directly measurable through typicality ratings, but can be captured with indirect methods such as eye movements during reading. The cognitive dissociation between these two factors is evident in the results of Experiment 2, where items possessed a low degree of gender typicality. Based on explicit ratings, the roles (e.g., manager, politician) were not classified as gender-typical, but they still triggered a mismatch effect in the eye-tracking measures, due to an automatic association of the professional role with a gender stereotype. Therefore, we can conclude that the concept of gender typicality could actually be split into two cognitive components: an explicit one, which can be recorded through classical typicality ratings and corresponds to beliefs on the distribution of men and women in a specific field, and an automatic one, which is revealed with indirect methods and is stored in readers' long-term memory together with the semantics of the respective role.

Furthermore, a cross-linguistic comparison with studies on grammatical gender languages suggests that the presence or absence of a grammatical gender system in the investigated language may play a key role in the processing of gender typicality cues, even when morphological/grammatical gender cues are not present in the text, but only cognitively available to the reader. More specifically, we argue that a grammatical gender system may make gender typicality cues more salient in comparison to a natural gender language. This is, however, open to debate [cf. Irmen and Rossberg, 2004; Gygax et al., 2008, on the relation between gender typicality and grammatical gender]. In a study employing a picture categorization paradigm in Italian and Spanish, Cubelli et al. (2011) show that grammatical gender is automatically activated, even if its retrieval is not required to accomplish the task. This consideration may suggest that gender information is already available in the cognitive representation of a reader possessing a grammatical gender system—even when no morphological markings are required for comprehension or presented in the stimuli—and trigger a faster processing of the gender mismatch.

Finally, a cross-linguistic comparison of the present study with grammatical gender language studies reveals a similar finding on the asymmetrical distribution of the gender mismatch effect, which had been previously reported only in studies on languages with a grammatical gender system (in Italian, Cacciari and Padovani, 2007; in German, Irmen et al., 2010). Specifically, pairwise contrasts in Experiment 1 revealed a significant effect in the condition of the masculine pronoun related to the incongruent female context, but no effect on the feminine pronoun related to the incongruent male context. In a study with event related potentials, Siyanova-Chanturia et al. (2012) document an N400-like effect for the masculine pronoun only, preceded by an incongruent typically female role noun (e.g., *insegnante-lui*). The N400 is assumed to represent a violation in semantic expectations, which is also at the basis of the gender mismatch asymmetry effect in eye movements. Our findings in English supports the cross-linguistic evidence that gender stereotypes may affect the processing of masculine and feminine anaphors differently. Socio-psychological theories on expectations related to gender roles may be required to explain this effect, as it may not only be due to the features of a particular gender system. However, further comparative studies and replications are necessary to determine the exact role of the gender system of a reader's language on the interpretation of gender-typical cues and its interaction with the process of anaphor resolution.

### Conflict of interest statement

The authors declare that the research was conducted in the absence of any commercial or financial relationships that could be construed as a potential conflict of interest.

## Footnotes

^1^We are grateful to Chuck Clifton for making the software available on the web page http://www.psych.umass.edu/eyelab/ (eye-tracking lab of the University of Massachusetts, UMass at Amherst, USA).

^2^Participants extended both arms and created an opening with their hands, through which they fixated a point on the wall. Then they slowly moved their hands toward their eyes, while fixating the point through the opening. At a close distance, in order to continue to fixate the point, the opening was drawn either in front of the left or the right eye, according to ocular dominance.

^3^Estimates obtained from the fitted models represent the model's prediction and take the crossed random effects into consideration. Therefore, values reported in the text may differ from the aggregated means reported in the tables.

^4^In first fixation time and first pass time, the first region of the target sentence was discarded from the analysis because of high percentage of missing values (33.5%) in comparison to the average skipping rate (17.4%). The high skipping rate of the first region may be explained by the fact that this region is represented by a short temporal adverb (e.g., “Today”) which may be easily skipped in early reading stages.

## References

[B1] Baayen R. H. Davidson D. J. Bates D. M. (2008). Mixed-effects modeling with crossed random effects for subjects and items. J. Mem. Lang. 59, 390–412. 10.1016/j.jml.2007.12.005

[B2] Bates D. Mächler M. Bolker B. Walker S. (2014). Fitting linear mixed-effects models using lme4. arXiv preprint arXiv:1406.5823. 24935795

[B3] Boland J. E. (2004). Linking eye movements to sentence comprehension in reading and listening, in The Online Study of Sentence Comprehension, eds Carreiras M. Clifton C. Jr (New York, NY: Psychology Press), 51–76.

[B4] Cacciari C. Corradini P. Padovani R. Carreiras M. (2011). Pronoun resolution in Italian: the role of grammatical gender and context. J. Cogn. Psychol. 23, 416–434. 10.1080/20445911.2011.526599

[B5] Cacciari C. Padovani R. (2007). Further evidence on gender stereotype priming in language: semantic facilitation and inhibition on Italian role nouns. Appl. Psycholinguist. 28, 277–293. 10.1017/S0142716407070142

[B6] Cubelli R. Paolieri D. Lotto L. Job R. (2011). The effect of grammatical gender on object categorization. J. Exp. Psychol. Learn. Mem. Cogn. 37, 449–460. 10.1037/a0021965 21261427

[B7] Duffy S. A. Keir J. A. (2004). Violating stereotypes: eye-movements and comprehension processes when text conflicts with world knowledge. Mem. Cogn. 32, 551–559. 10.3758/BF03195846 15478749

[B8] Esaulova Y. Reali C. von Stockhausen L. (2014). Influences of grammatical and stereotypical gender during reading: eye movements in pronominal and noun phrase anaphor resolution. Lang. Cogn. Neurosci. 29, 781–803. 10.1080/01690965.2013.794295

[B9] Gabriel U. Gygax P. Sarrasin O. Garnham A. Oakhill J. (2008). Au pairs are rarely male: norms on the gender perception of role names across English, French, and German. Behav. Res. Methods 40, 206–212. 10.3758/BRM.40.1.206 18411544

[B10] Garnham A. Oakhill J. Reynolds D. (2002). Are inferences from stereotyped role names to characters'gender made elaboratively? Mem. Cogn. 30, 439–446. 10.3758/BF03194944 12061764

[B11] Gygax P. Gabriel U. Sarrasin O. Garnham A, Oakhill, J. (2008). Generically intended, but specifically interpreted: when beauticians, musicians, and mechanics are all men. Lang. Cogn. Neurosci. 23, 464–485. 10.1080/01690960701702035

[B12] Hellinger M. Bußmann H. (2001). Gender Across Languages: The Linguistic Representation of Women and Men, Vol. 1 Amsterdam: Benjamins.

[B13] Irmen L. (2007). What's in a (role) name? Formal and conceptual aspects of comprehending personal nouns. J. Psycholinguist. Res. 36, 431–456. 10.1007/s10936-007-9053-z 17372839

[B14] Irmen L. Holt D.V. Weisbrod M. (2010). Effects of role typicality on processing person information in German: evidence from an ERP study. Brain Res. 1353, 133–144. 10.1016/j.brainres.2010.07.018 20637743

[B15] Irmen L. Rossberg N. (2004). Gender markedness of language: the impact of grammatical and nonlinguistic information on the mental representation of person information. J. Lang. Soc. Psychol. 23, 272–307. 10.1177/0261927X04266810

[B16] Irmen L. Schumann E. (2011). Processing grammatical gender of role nouns: further evidence from eye movements. J. Cogn. Psychol. 23, 998–1014. 10.1080/20445911.2011.596824

[B17] Jäger L. A. Benz L. Roeser J. Dillon B. W. Vasishth S. (2015). Teasing apart retrieval and encoding interference in the processing of anaphors. Front. Psychol. 6:506. 10.3389/fpsyg.2015.00506 26106337 PMC4460324

[B18] Kennison S. M. Trofe J. L. (2003). Comprehending pronouns: a role for word-specific gender stereotype information. J. Psycholinguist. Res. 32, 355–378. 10.1023/A:1023599719948 12845944

[B19] Kreiner H. Sturt P. Garrod G. (2008). Processing definitional and stereotypical gender in reference resolution: evidence from eye-movements. J. Mem. Lang. 58, 239–261. 10.1016/j.jml.2007.09.003

[B20] Kuznetsova A. Brockhoff P.B. Christensen R.H.B. (2013). LmerTest: Tests for Random and Fixed Effects for Linear Mixed Effect Models (Lmer Objects of lme4 Package). R package version 2.0-0. Available online at: http://CRAN.Rproject.org/package=lmerTest/

[B21] Oakhill J. Garnham A. Reynolds D. (2005). Immediate activation of stereotypical gender information. Mem. Cogn. 33, 972–983. 10.3758/BF03193206 16496719

[B22] Osterhout L. Bersick M. McLaughlin J. (1997). Brain potentials reflect violations of gender stereotypes. Mem. Cogn. 25, 273–285. 10.3758/BF03211283 9184479

[B23] Osterhout L. Mobley L. (1995). Event-related brain Potentials elicited by failure to agree. J. Mem. Lang. 34, 739–773. 10.1006/jmla.1995.1033

[B24] Pyykkönen P. Hyönä J. van Gompel R. P. (2010). Activating gender stereotypes during online spoken language processing. Exp. Psychol. 57, 126–133. 10.1027/1618-3169/a000016 20178931

[B25] Reali C. Esaulova Y. von Stockhausen L. (2015). Isolating stereotypical gender in a grammatical gender language: evidence from eye movements. Appl. Psycholinguist. 36, 977–1006. 10.1017/S0142716414000010

[B26] R Core Team (2012). R: A Language and Environment for Statistical Computing. Vienna: The R Foundation. Available online at: http://www.R-project.org/

[B27] Sato S. Gygax P. Gabriel U. (2013). Gender inferences: grammatical features and their impact on the representation of gender in bilinguals. Bilingualism 16, 792–807. 10.1017/S1366728912000739

[B28] Siyanova-Chanturia A. Pesciarelli F. Cacciari C. (2012). The electrophysiological underpinnings of processing gender stereotypes in language. PLoS ONE 7:e48712. 10.1371/journal.pone.0048712 23226494 PMC3513306

[B29] Sturt P. (2003). The time-course of the application of binding constraints in reference resolution. J. Mem. Lang. 48, 542–562. 10.1016/S0749-596X(02)00536-3

