# Supplementary material for: Role descriptions induce gender mismatch effects in eye movements during reading
Source: Front Psychol. 2015 Nov 3;6:1607. doi: 10.3389/fpsyg.2015.01607 (PMC4630541; doi:10.3389/fpsyg.2015.01607)
Supplement: Supplementary file 1 [file Table1.DOC]

Table S1. Best fitting models detected by the *step*() function in lmerTest (lme4 package), separated for eye-tracking measure and region, for experiment 1.

**First fixation time**

***Region 1***

-

***Region 2***

Final model<- lmer(Logar_reg2 ~ (1 | part))

***Region 3***

Final model<- lmer(Logar_reg3 ~ (1 | part) + (1 | item))

***Region 4***

Final model<- lmer(Logar_reg4 ~ (1 | part))

**First pass fixation duration**

***Region 1***

-

***Region 2***

Final model<- lmer(Logar_reg2 ~ pron + typ + part_sex + L2 + (1 | part) + pron:typ + pron:part_sex + typ:part_sex + pron:typ:part_sex)

***Region 3***

Final model<- lmer(Logar_reg3 ~ pron + typ + part_sex + L3 + (1 | part) + (1 | item) + pron:typ + part_sex:L3)

***Region 4***

Final model<- lmer(Logar_reg4 ~ L4 + (1 | part) + (1 | item))

**Regression path**

***Region 1***

Final model<- lmer(Logar_reg1 ~ (1 | part) + (1 | item))

***Region 2***

Final model<- lmer(Logar_reg2 ~ pron + L2 + (1 | part) + (1 | item) + pron:L2)

***Region 3***

Final model<- lmer(Logar_reg3 ~ pron + part_sex + L3 + (1 | part) + (1 | item) + part_sex:L3)

***Region 4***

Final model<- lmer(Logar_reg4 ~ L4 + (1 | part) + (1 | item))

**Total time**

***Region 1***

Final model<- lmer(Logar_reg1 ~ L1 + (1 | part) + (1 | item))

***Region 2***

Final model<- lmer(Logar_reg2 ~ pron + typ + part_sex + L2 + (1 | part) + (1 | item) + pron:typ + pron:part_sex + typ:part_sex + pron:L2 + typ:L2 + part_sex:L2 + pron:typ:part_sex +pron:typ:L2 + pron:part_sex:L2 + typ:part_sex:L2 + pron:typ:part_sex:L2)

***Region 3***

Final model<- lmer(Logar_reg3 ~ pron + typ + L3 + (1 | part) + (1 | item) + pron:typ + pron:L3 + typ:L3 + pron:typ:L3)

***Region 4***

Final model<- lmer(Logar_reg4 ~ L4 + (1 | part) + (1 | item))

**Regressions into the region**

***Region 1***

Final model<- lmer(Logar_reg1 ~ pron + typ + part_sex + (1 | part) + (1 | item) + pron:typ + pron:part_sex + typ:part_sex + pron:typ:part_sex)

***Region 2***

Final model<- lmer(Logar_reg2 ~ pron + part_sex + (1 | part) + (1 | item) + pron:part_sex)

***Region 3***

Final model<- lmer(Logar_reg3 ~ (1 | part) + (1 | item))

***Region 4***

_

**Regressions out of the region**

***Region 1***

_

***Region 2***

Final model<- lmer(Logar_reg2 ~ pron + typ + part_sex + (1 | part) + (1 | item) + pron:typ + pron:part_sex + typ:part_sex + pron:typ:part_sex)

***Region 3***

Final model<- lmer(Logar_reg3 ~ (1 | part) + (1 | item))

***Region 4***

Final model<- lmer(Logar_reg4 ~ pron + typ + (1 | part) + (1 | item) + pron:typ)

**Best fitting models detected by the *step*() function of lmerTest (lme4 package), separated for eye-tracking measure and region, for experiment 2.**

**First fixations**

***Region 1***

Final model<- lmer(Logar_reg1 ~ pron + typ + part_sex + L1 + (1 | part) + pron:part_sex + typ:part_sex + pron:L1 + typ:L1 + part_sex:L1 + pron:part_sex:L1 + typ:part_sex:L1)

***Region 2***

Final model<- lmer(Logar_reg2 ~ typ + part_sex + L2 + (1 | part) + typ:L2 + part_sex:L2)

***Region 3***

Final model<- lmer(Logar_reg3 ~ (1 | part) + (1 | item))

***Region 4***

Final model<- lmer(Logar_reg4 ~ L4 + (1 | part))

**First pass fixation duration**

***Region 1***

Final model<- lmer(Logar_reg1 ~ typ + part_sex + L1 + (1 | part) + (1 | item) + typ:part_sex + typ:L1 + part_sex:L1 + typ:part_sex:L1)

***Region 2***

Final model<- lmer(Logar_reg2 ~ pron + typ + part_sex + L2 + (1 | part) + (1 | item) + pron:typ + pron:part_sex + typ:part_sex + pron:L2 + typ:L2 + pron:typ:L2)

***Region 3***

Final model<- lmer(Logar_reg3 ~ part_sex + L3 + (1 | part) + (1 | item) + part_sex:L3)

***Region 4***

Final model<- lmer(Logar_reg4 ~ pron + typ + part_sex + L4 + (1 | part) + (1 | item) + pron:typ + pron:part_sex + typ:part_sex + pron:typ:part_sex)

**Regression path**

***Region 1***

Final model<- lmer(Logar_reg1 ~ L1 (1 | part) + (1 | item))

***Region 2***

Final model<- lmer(Logar_reg2 ~ part_sex + L2 + (1 | part) + (1 | item) + part_sex:L2)

***Region 3***

Final model<- lmer(Logar_reg3 ~ (1 | part) + (1 | item))

***Region 4***

Final model<- lmer(Logar_reg4 ~ L4 + (1 | part))

**Total time**

***Region 1***

Final model<- lmer(Logar_reg1 ~ pron + typ + part_sex + L1 + (1 | part) + (1 | item) + pron:typ + pron:part_sex + typ:part_sex + pron:L1 + typ:L1 + part_sex:L1 + pron:typ:part_sex + pron:typ:L1 + pron:part_sex:L1 + typ:part_sex:L1 + pron:typ:part_sex:L1)

***Region 2***

Final model<- lmer(Logar_reg2 ~ part_sex + L2 + (1 | part) + (1 | item) + part_sex:L2)

***Region 3***

Final model<- lmer(Logar_reg3 ~ typ + L3 + (1 | part) + (1 | item) + typ:L3)

***Region 4***

Final model<- lmer(Logar_reg4 ~ pron + typ + part_sex + L4 + (1 | part) + (1 | item) + pron:typ + pron:part_sex + typ:part_sex + pron:typ:part_sex)

**Regressions into the region**

***Region 1***

Final model<- lmer(Logar_reg1 ~ pron + typ + (1 | part) + (1 | item) + pron:typ)

***Region 2***

Final model<- lmer(Logar_reg2 ~ (1 | part) + (1 | item))

***Region 3***

Final model<- lmer(Logar_reg3 ~ (1 | part) + (1 | item))

***Region 4***

_

**Regressions out of the region**

***Region 1***

_

***Region 2***

Final model<- lmer(Logar_reg2 ~ (1 | part) + (1 | item))

***Region 3***

Final model<- lmer(Logar_reg3 ~ (1 | part) + (1 | item))

***Region 4***

Final model<- lmer(Logar_reg4 ~ typ + (1 | part) + (1 | item))
