# Supplementary material for: Role descriptions induce gender mismatch effects in eye movements during reading
Source: Front Psychol. 2015 Nov 3;6:1607. doi: 10.3389/fpsyg.2015.01607 (PMC4630541; doi:10.3389/fpsyg.2015.01607)
Supplement: Supplementary file 2 [file Table2.DOCX]

Table S2. Typicality ratings for Experiment 1

| Role Noun | Role Description | Pretest  Role noun | | Pretest  Description | | Follow-up Description | |
| --- | --- | --- | --- | --- | --- | --- | --- |
| MALE TYPICALITY | | *Mean* | (*SD*) | *Mean* | (*SD*) | *Mean* | (*SD*) |
| Construction worker | A. W. erects walls, uses mortar  and concrete. | 1.5 | (0.5) | 1.5 | (0.7) | 2.0 | (0.9) |
| Plumber | C. M. lays pipes, repairs burst pipes, maintains the sewage system. | 1.5 | (0.6) | 1.4 | (0.7) | 1.8 | (0.9) |
| Blacksmith | F. V. shapes metal, uses a hammer and anvil, works on hot iron. | 1.7 | (0.8) | 1.4 | (0.7) | 1.9 | (0.9) |
| Janitor | H. L. takes care of a building, carries out small repairs, keeps the keys. | 1.9 | (0.7) | 1.8 | (0.8) | 2.6 | (1.0) |
| Electrician | J. P. installs power lines and cables, checks electricity voltage. | 1.9 | (0.6) | 1.7 | (0.7) | 2.3 | (0.9) |
| Mechanic | K. L repairs cars and engines, checks brakes in a workshop. | 2.0 | (0.5) | 1.6 | (0.7) | 2.0 | (0.8) |
| Soldier | L. T. serves in the army in times of war, fires at command. | 2.0 | (0.5) | 1.4 | (0.7) | 2.1 | (1.2) |
| Carpenter | M. F. repairs and produces furniture, works with wood. | 2.0 | (0.5) | 2.2 | (0.9) | 2.6 | (0.9) |
| IT-expert | P. K. develops software, monitors computer systems. | 2.1 | (0.6) | 2.3 | (0.8) | 2.6 | (0.9) |
| Astronaut | R. B. travels into space in a rocket, works at NASA. | 2.2 | (0.7) | 2.4 | (0.8) | 2.8 | (0.9) |
| Engineer | R. F. has a technical university degree, designs machines and construction plans. | 2.5 | (0.6) | 2.3 | (0.9) | 2.6 | (1.1) |
| Train driver | V. M. drives a train, works for the railway. | 2.5 | (0.9) | 2.4 | (0.9) | 2.5 | (0.9) |
| FEMALE TYPICALITY | |  |  |  |  |  |  |
| Cashier | W. A. works in a supermarket, scans items at the check-out counter. | 5.5 | (0.7) | 5.3 | (1.0) | 4.5 | (0.9) |
| Nutritionist | V. F. draws up plans for a healthy diet, helps people to lose weight. | 5.5 | (0.9) | 5.4 | (1.3) | 4.7 | (0.9) |
| Florist | I. R. sells flowers, makes up bouquets in a shop. | 5.8 | (0.5) | 6.0 | (0.9) | 5.7 | (1.0) |
| Flight attendant | K. P. works for Lufthansa, assists passengers, gives safety instructions. | 5.8 | (0.6) | 5.8 | (1.2) | 4.6 | (1.0) |
| School teacher | B. R. teaches at a primary school, instructs children in reading. | 5.8 | (0.7) | 5.8 | (0.7) | 4.9 | (1.0) |
| Fortune teller | I. R. predicts the future, uses a crystal ball. | 5.9 | (1.0) | 6.0 | (1.3) | 5.6 | (1.0) |
| Babysitter | L. H. takes care of other people´s children in the evenings. | 5.9 | (0.8) | 6.0 | (0.8) | 5.5 | (0.9) |
| Clinic receptionist | T. L. works in a clinic at the front desk, organizes appointments. | 5.9 | (1.0) | 6.2 | (1.3) | 4.9 | (0.8) |
| Kindergarten teacher | M. V. teaches two- to five-year-old children, does handicrafts and tells stories. | 6.1 | (0.6) | 6.2 | (1.2) | 5.4 | (1.1) |
| Secretary | L. K. makes appointments, deals with the correspondence in an office. | 6.2 | (0.7) | 6.1 | (1.2) | 4.8 | (0.9) |
| Midwife/ Obstetrician | M. C. assists in childbirths, works at a hospital. | 6.3 | (0.6) | 6.6 | (0.7) | 5.7 | (0.9) |
| Beautician | P. J. does clients´ make up, plucks eyebrows, removes hair. | 6.5 | (0.6) | 6.4 | (0.9) | 6.1 | (1.0) |
| NEUTRAL TYPICALITY | |  |  |  |  |  |  |
| Skier | B. T. practices winter sports, participates in slalom races. | 3.8 | (0.6) | 3.6 | (0.9) | 3.7 | (0.9) |
| Swimmer | D. K. exercises in the water, wears a swimsuit and goggles. | 3.9 | (0.3) | 4.3 | (1.1) | 4.9 | (1.1) |
| Doctor | E. M. studied medicine, makes diagnoses, cures illnesses. | 3.9 | (0.6) | 3.8 | (0.5) | 3.8 | (0.7) |
| Artist | F. H. is creative, paints and makes sculptures. | 4.0 | (0.6) | 4.1 | (0.9) | 4.5 | (1.0) |
| Intern | J. M. tries a job for a short period of time, gains experience on the job. | 4.0 | (0.4) | 3.9 | (0.9) | 3.9 | (0.8) |
| Writer | K. W. writes books, sometimes gives readings. | 4.0 | (0.2) | 4.1 | (0.7) | 4.2 | (0.9) |
| Pensioner | M. N. is finished with working life, receives a pension. | 4.1 | (0.5) | 3.8 | (0.8) | 3.9 | (0.7) |
| Musician | K. M. plays an instrument professionally in an orchestra. | 4.1 | (0.3) | 3.8 | (0.7) | 4.2 | (0.9) |
| Student | R. S. studies at the university, attends lectures and exams. | 4.1 | (0.3) | 3.8 | (0.7) | 4.1 | (0.5) |
| Actor | J. W. plays different roles on the stage or in movies. | 4.1 | (0.3) | 4.2 | (0.4) | 4.1 | (0.6) |
| Singer | R. T. performs at concerts and sings in the opera. | 4.2 | (0.5) | 4.4 | (1.0) | 4.6 | (0.9) |
| Pharmacist | S. L. studied pharmacy, sells medicine in a drugstore. | 4.3 | (0.8) | 4.7 | (1.2) | 4.0 | (0.7) |
