# Supplementary material for: Role descriptions induce gender mismatch effects in eye movements during reading
Source: Front Psychol. 2015 Nov 3;6:1607. doi: 10.3389/fpsyg.2015.01607 (PMC4630541; doi:10.3389/fpsyg.2015.01607)
Supplement: Supplementary file 3 [file Table3.DOCX]

Table S3. Typicality ratings for Experiment 2

| Role Noun | Role Description | Pretest  Role noun | | | Follow-up Description | | |
| --- | --- | --- | --- | --- | --- | --- | --- |
| SLIGHTLY MALE TYPICALITY | | *Mean* | | (*SD*) | *Mean* | (*SD*) | |
| Bartender | | X mixes cocktails, juggles with the bottles. | 2.8 | (1.1) | | 3.2 | (1.0) |
| Politician | | X refuses to admit mistakes in front of the voters. | 2.8 | (0.9) | | 2.9 | (1.3) |
| Researcher | | X constantly works on new research ideas. | 2.9 | (0.9) | | 3.9 | (0.6) |
| Lifeguard | | X saves people from drowning at the swimming pool. | 2.9 | (1.1) | | 3.4 | (0.8) |
| Manager | | X is responsible for a factory, has to work overtime. | 2.9 | (0.7) | | 2.7 | (0.9) |
| Cook | | X works in a restaurant, prepares sophisticated dishes. | 2.9 | (0.7) | | 3.6 | (0.8) |
| Postman | | X delivers the mail every day in all kinds of weather. | 3.0 | (1.0) | | 2.8 | (1.1) |
| Police officer | | X works at the police station, knows the frequency of robberies. | 3.0 | (1.0) | | 3.4 | (1.0) |
| Destist | | X often treats severe cases of tooth decay. | 3.0 | (0.8) | | 3.6 | (1.1) |
| Secret agent | | X accomplishes missions, spies on foreign governments. | 3.2 | (0.9) | | 3.3 | (1.1) |
| Lawyer | | X earned a degree in law after many years of study. | 3.2 | (0.8) | | 3.9 | (0.8) |
| Marathon runner | | X exercises every day, covers 42 km in one training run. | 3.3 | (0.7) | | 3.4 | (1.0) |
| SLIGHTLY FEMALE TYPICALITY | | |  |  | |  |  |
| Designer | | X prepares sketches of dresses, artefacts, and furniture. | 4.6 | (0.9) | | 5.1 | (1.1) |
| Physio-therapist | | X looks after patients during rehabilitation. | 4.6 | (0.7) | | 4.7 | (0.8) |
| Masseuse | | X gives massages, relieves muscular tension and pain. | 4.6 | (0.9) | | 4.7 | (1.1) |
| Waiter/ress | | X serves drinks, brings food in a café. | 4.6 | (0.9) | | 4.6 | (1.0) |
| Psychologist | | X studies human thinking and behaviour. | 4.9 | (0.9) | | 4.6 | (0.7) |
| Translator | | X translates and speaks many different languages. | 5.0 | (0.7) | | 4.3 | (0.7) |
| Nurse | | X changes bedsheets, measures temperature of patients. | 5.1 | (0.8) | | 5.2 | (0.9) |
| Telephonist | | X receives calls from many customers at the call-center. | 5.1 | (0.8) | | 4.1 | (0.9) |
| Flutist | | X plays the flute professionally in the city orchestra. | 5.2 | (1.1) | | 4.7 | (1.2) |
| Cleaner | | X cleans every day public and private buildings. | 5.3 | (1.4) | | 4.3 | (0.9) |
| Aerobic instructor | | X teaches several aerobic courses in a gym. | 5.3 | (1.0) | | 5.1 | (0.9) |
| Hairdresser | | X cuts and styles professionally people's hair. | 5.4 | (0.6) | | 4.8 | (1.0) |
| NEUTRAL TYPICALITY | | |  |  | |  |  |
| Skier | | B. T. practices winter sports, participates in slalom races. | 3.8 | (0.6) | | 3.7 | (0.9) |
| Swimmer | | D. K. exercises in the water, wears a swimsuit and goggles. | 3.9 | (0.3) | | 4.9 | (1.1) |
| Doctor | | E. M. studied medicine, makes diagnoses, cures illnesses. | 3.9 | (0.6) | | 3.8 | (0.7) |
| Artist | | F. H. is creative, paints and makes sculptures. | 4.0 | (0.6) | | 4.5 | (1.0) |
| Intern | | J. M. tries a job for a short period of time, gains experience on the job. | 4.0 | (0.4) | | 3.9 | (0.8) |
| Writer | | K. W. writes books, sometimes gives readings. | 4.0 | (0.2) | | 4.2 | (0.9) |
| Pensioner | | M. N. is finished with working life, receives a pension. | 4.1 | (0.5) | | 3.9 | (0.7) |
| Musician | | K. M. plays an instrument professionally in an orchestra. | 4.1 | (0.3) | | 4.2 | (0.9) |
| Student | | R. S. studies at the university, attends lectures and exams. | 4.1 | (0.3) | | 4.1 | (0.5) |
| Actor | | J. W. plays different roles on the stage or in movies. | 4.1 | (0.3) | | 4.1 | (0.6) |
| Singer | | R. T. performs at concerts and sings in the opera. | 4.2 | (0.5) | | 4.6 | (0.9) |
| Pharmacist | | S. L. studied pharmacy, sells medicine in a drugstore. | 4.3 | (0.8) | | 4.0 | (0.7) |
